# Hypovitaminosis D3, Leukopenia, and Human Serotonin Transporter Polymorphism in Anorexia Nervosa and Bulimia Nervosa

**DOI:** 10.1155/2016/8046479

**Published:** 2016-01-24

**Authors:** Anna Tasegian, Francesco Curcio, Laura Dalla Ragione, Francesca Rossetti, Samuela Cataldi, Michela Codini, Francesco Saverio Ambesi-Impiombato, Tommaso Beccari, Elisabetta Albi

**Affiliations:** ^1^Department of Pharmaceutical Sciences, University of Perugia, Via Fabretti No. 48, 06123 Perugia, Italy; ^2^Department of Clinical and Biological Sciences, University of Udine, Piazzale Kolbe No. 4, 33100 Udine, Italy; ^3^Department of Eating Disorder, Palazzo Francisci Todi, USL 1 Umbria, Via Cesia No. 65, 06059 Todi, Italy

## Abstract

Vitamin D3 has been described to have different extraskeletal roles by acting as parahormone in obesity, diabetes, cancer, cognitive impairment, and dementia and to have important regulatory functions in innate immunity. There are no studies showing extraskeletal changes associated with hypovitaminosis D3 in eating disorders.* Methods.* We have analyzed the blood of 18 patients affected by anorexia nervosa and bulimia nervosa collected over a 15-month period. We performed a panel of chemical and clinical analyses: the assay of vitamin D3, the immunoblotting of vitamin D receptor and peroxisome proliferator-activated receptor gamma, and the genotyping of 5-hydroxytryptamine transporter linked polymorphic region.* Results.* We choose 18 patients with a normal blood test profile such as thyroid hormones, hepatic and renal parameters, triglycerides, proteins, vitamin B12, and folic acid. Among these emerged the case of a woman with long-term anorexia nervosa and the case of a woman with long-term bulimia nervosa both complicated by anxiety and depression, severe hypovitaminosis D3, decrease of vitamin D receptor, leukopenia, and 5-hydroxytryptamine transporter linked polymorphic region short allele.* Conclusion.* The results induce hypothesising that the severe hypovitaminosis D3 might be responsible for the lack of the inflammatory response and the depressive symptoms in patients with long-term eating disorders.

## 1. Introduction

The eating disorders, such as anorexia nervosa (AN) and bulimia nervosa (BN), have been classically described with a clear female preponderance [1]. AN is a psychopathology characterized by the relentless drive for thinness and/or a morbid fear of fatness and BN is characterized by recurrent episodes of overeating in which large amounts of food are consumed in short periods [2]. Both are often accompanied by comorbid psychiatric disorders, that is, affective, anxiety, and personality disorders [2]. Eating disorders have multifactorial etiology, from endocrine abnormalities to genetic, psychological, and environment factors [3]. Individuals with AN and BN are consistently characterized by perfectionism, obsessive-compulsiveness, and dysphoric mood [4].

Vitamin D3 is recognized to have different extraskeletal roles by acting as parahormone. Recent evidence correlates serum vitamin D3 deficiency to obesity, diabetes, cardiovascular risks, cancer [5], cognitive impairment, and dementia [6]. The anti-inflammation activity of vitamin D3 has drawn more and more attention of researchers to investigate its role in regulating the progression of inflammatory diseases [7] as an important regulator of innate immunity [8]. Vitamin D3 deficiency has been associated with inflammatory bowel disease [9], chronic kidney disease [10], asthma, and atopy [11]. Until now vitamin D3 deficiency in patients with eating disorders was correlated only with the risk of osteoporosis [12, 13]. It is known that vitamin D3 modulates peroxisome proliferator-activated receptor gamma (PPAR*γ*) [14], molecule involved in inflammation related to the diet [15, 16].

Today is growing acknowledgement that serotonin (5-hydroxytryptamine, 5-HT) variations make a substantial contribution to the pathogenesis of AN and BN. Neuroendocrine rhythms, including food intake, sleep, and reproductive activity together with mood, emotion, and cognition, are regulated by the midbrain raphe 5-HT system [17]. 5-HT is a monoamine neurotransmitter synthesized in the presynaptic neuron and released, during neurotransmission process, by presynaptic vesicles into the synaptic cleft; then it binds to receptors on the postsynaptic neuron where it is metabolised by the monoamine oxidase A enzyme. The 5-HT transporter (5-HTT) resides in the presynaptic membrane allowing reuptake of excess hormone from the synaptic cleft [18]. Relationship between 5-HT dysfunction and dysregulation of eating behaviour, mood, and general psychopathology was widely reported in AN and BN [4, 19, 20]. 5-HTT function has impact on early brain development, event-related synaptic plasticity, depression, and bipolar, anxiety, obsessive-compulsive, schizophrenic, and eating disorders and it is a prime target for widely used antidepressants [17]. 5-HTT polymorphism is generated by a 44 bp deletion and involves repeat units 6–8, and it is located close to a “hot spot” for deletion mutagenesis (TGCAGC) in the linked polymorphic region; allelic variation in 5-HTT gene plays a role in the expression and modulation of complex traits and behaviour [17]. Associations between 5-HTT-linked polymorphic region (5-HTTLPR) variations and eating-disorder subphenotypes were demonstrated [21]. In this way 5-HTTLPR deleted (S) allele seems to represent a risk factor for eating disorders, especially for AN [22]. Patrick and Ames reported a regulation of 5-HT synthesis by vitamin D3 in patients with autism [23], attention deficit hyperactivity disorder, bipolar disorder, schizophrenia, and impulsive behaviour [24].

The aim of the work was to highlight the association among severe hypovitaminosis D3, reduction of vitamin D receptor (VDR), leukopenia, and 5-HTTLPR in patients with long-term AN and BN.

## 2. Methods

### 2.1. Ethics Statement

All participants provided written informed consent prior to inclusion in this project and were treated in accordance with the Declaration of Helsinki. The study protocol and process were assessed and approved by the Ethics Committee of the Aziende Sanitarie (CEAS) della Regione Umbria, Italy.

### 2.2. Patients

Blood samples of 18 patients affected by AN (n. 11) and/or BN (n. 7) were collected over a 15-month period (October 2014–January 2015) from the Department of Eating Disorder, Palazzo Francisci (USL 1 Umbria, Todi, Italy). The population consisted of all females; average age was 40 years (range 14–65 yrs). The chemical-clinic analyses were performed in the diagnostic laboratory of Todi (USL 1 Umbria, Italy). Whole blood, centrifuged cells, and serum were stored at −20°C until used for the assay of vitamin D3, immunoblotting, and genotyping analysis.

### 2.3. Materials

Standard vitamin D3 was obtained from Sigma Chemical Co. (St. Louis, Missouri, USA); anti-peroxisome proliferator-activated receptor gamma (PPAR*γ*) and anti-vitamin D receptor (VDR) antibodies were obtained from Santa Cruz Biotechnology, Inc. (California, USA).

### 2.4. Clinical Measures

Participants' weight and height were evaluated. The levels of thyroid hormones, hemochrome, hepatic and renal parameters, iron, ferritin, cholesterol, triglycerides, proteins, vitamin B12, and folic acid were measured.

### 2.5. Serum Assays for Vitamin D3

Serum vitamin D3 levels were measured by LC/MS/MS method. Before the analysis, the sample was prepared by adding 150 *μ*L of precipitation reagent, containing Internal Standard, to 50 *μ*L of patient serum or quality control serum; after 10 minutes of incubation, 50 *μ*L of supernatant was injected. The signal from the analytes was measured against the calibration curve using the MultiQuant 2.1 software. For the analysis, Shimadzu UFLC-XR system, Shimadzu, Kyoto, Japan, equipped with a DGU-20A3 degasser, LC-20AD XR binary pump, SIL-20ACXR autosampler, CTO-20AC column oven, and a communication bus module CBM-20A was used. The UFLC system was connected to a 4000 QTRAP linear ion trap mass spectrometer (AB Sciex, Concord, ON, Canada) equipped with an APCI source operated in the positive ionization mode. A 6-port automatic switching valve was interposed between the chromatographic system and the mass spectrometer. The controller software was Analyst version 1.6. The limit of detection (LLOD) for this assay was 0.67 *μ*g/L. The low limit of quantification (LLOQ) was 2.25 *μ*g/L, and the linearity was 2.25–250 *μ*g/L. CVs were 6% at various concentrations across the analytical measurement range.

### 2.6. Electrophoresis and Western Blot Analysis

The analysis was performed as previously reported [25]. 30 *μ*g of protein was used for SDS-PAGE electrophoresis in 10% polyacrylamide slab gel. Proteins were transferred into nitrocellulose for 90 min, and the membranes were blocked for 30 min with 0.5% no fat-dry milk in PBS, pH 7.5, and incubated overnight at 4°C with antibody anti-PPAR*γ* or VDR. The blots were incubated with horseradish-conjugated secondary antibodies for 90 min. The enhanced chemiluminescence (ECL) reaction was performed with the ECL kit from Amersham (Rainham, Essex, UK). Immunoblots of proteins were reprobed after stripping. The apparent molecular weight of the proteins was calculated according to the migration of molecular size standard. The area density of the bands was evaluated by densitometry scanning and analyzed with Scion Image.

### 2.7. Genotyping of 5-HTTLPR

Genomic DNA (gDNA) was extracted from whole blood samples using QIAamp® DNA Mini kit (QIAGEN). Genotyping of the 5-HTTLPR polymorphism was performed by polymerase chain reaction using the Phusion Hot Start II High Fidelity DNA Polymerase (Thermo Scientific). The following primer pair was used: 5′-GGCGTTGCCGCTCTGAATGC-3′ (primer forward), 5′-GAGGGACTGAGCTGGACAACCAC-3′ (primer reverse) [26, 27]. The amplification of ~50 ng of gDNA was carried out after 30′′ of initial denaturation at 98°C for 35 cycles (denaturation at 98°C for 15′′, annealing at 69°C for 30′′, and extention at 72°C for 15′′) followed by a final extention at 72°C for 5′. 10 *μ*L aliquots of each PCR product were resolved on 2.5% agarose gel electrophoresis in 1x TBE buffer at 90 Volts. Genotype was determined by fragment sizes: 484 bp (S, short allele) and 528 bp (L, long allele). 100 bp SHARPMASS (Euroclone) was used as DNA ladder.

### 2.8. Statistical Analysis

Three experiments performed in duplicate were performed for each analysis. Data are expressed as mean ± SD and *t*-test was used for statistical analysis.

## 3. Results and Discussion

### 3.1. Results

AN (n. 11) and BN (n. 7) patients were selected to have normal blood test profile as thyroid hormones, hepatic and renal parameters, triglycerides, proteins, vitamin B12, and folic acid. Thus 18 patients were enrolled.

To highlight a possible association with immunity cells and to exclude defects of intestinal absorption and/or of metabolic disorders, the concentration of vitamin D3 was correlated to the total white blood cells (WBC), neutrophils (N), lymphocytes (L), iron, ferritin, transferrin, cholesterol, and glycemia (Table 1). All patients had normal levels of ferritin, transferrin, and glycemia, and only 3 patients showed low levels of iron and 1 patient low level of cholesterol, without changes of vitamin D.

Of 18 patients, 13 presented normal levels of vitamin D3, between 16 and 60 ng/mL [28] (group A) and 5 patients were vitamin D3-deficient. We chose to divide patients with low levels of vitamin D3 in 2 groups: patients with 10–16 ng/mL vitamin D3 (group B, patients 1, 5, and 6) and patients with 1–4 ng/mL (group C, patients 13, 16).

The patients of groups A and B showed no changes in WBC; only patient n. 2 presented reduction of N and increase of L as a sign of possible chronic inflammation (Table 1). Interestingly, patients of group C had low level of total WBC, patient 13 low level of N (1.59 × 10^3^), and patient 16 low level of L (0.71 × 10^3^). These data could suggest, therefore, the existence of a relation between the low level of vitamin D3 and the WBC disorders.

**Figure 1:**
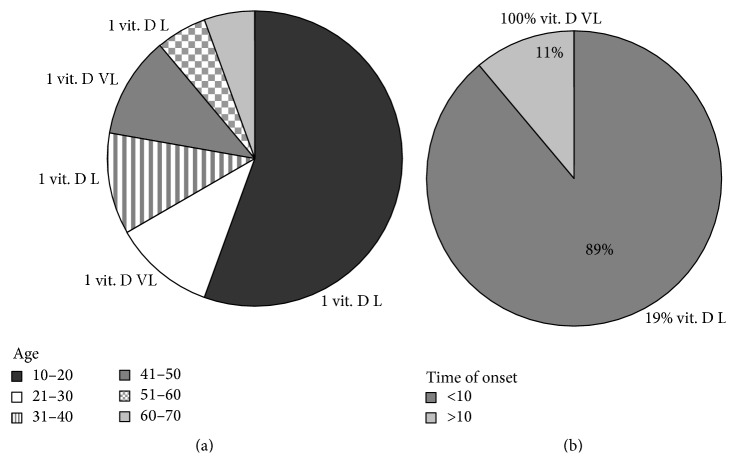
Level of vitamin D3 in patients affected with anorexia nervosa and bulimia nervosa. The data were analysed in relation to (a) the age and (b) time of the onset. Vit. D L, low level vitamin D3; Vit. D VL, very low level of vitamin D3.

It is important to note that there was no correlation between the concentration of vitamin D3 and age. In fact, of 18 patients 10 were found to have an age in the range of 10–20 years, 2 in the range of 21–30 years, 2 in the range of 31–40 years, and 2 in the range of 41–50 years, and 1 patient was found to have an age in the range of 51–61 years and 1 in the range of 61–70 years (Figure 1(a)). Patients with low concentration of vitamin D3 had 16, 40, and 55 years and patients with very low level of vitamin D3 had 29 and 44 years (Figure 1(a)).

Differently a relation between level of vitamin D3 and time of onset of the eating disorders was evident. In fact, of 18 patients 16 (89%) had a time of onset under 10 years and included patients with normal level and low level of vitamin D3, whereas 2 patients (11%) had a very long history of disease, one with AN (patient n. 13) and one with BN (patient n. 16), and both had very low level of vitamin D3 (Table 1 and Figure 1). The results suggested the importance of blood concentration of vitamin D3 in patients who had a very long history on eating disorders but remained unknown if these patients had also deficiency of VDR in the blood cells.

To address this question, the immunoblotting analysis was performed with a specific antibody against vitamin D3 receptor (VDR), in patients n. 13 (experimental 1, Ex1) and n. 16 (experimental 2, Ex2). Patient number 11 with normal level of vitamin D3 (control 1, C1) and patient number 5 with low level of vitamin D3 (control 2, C2), both with similar blood test profile, were chosen as controls.

**Figure 2:**
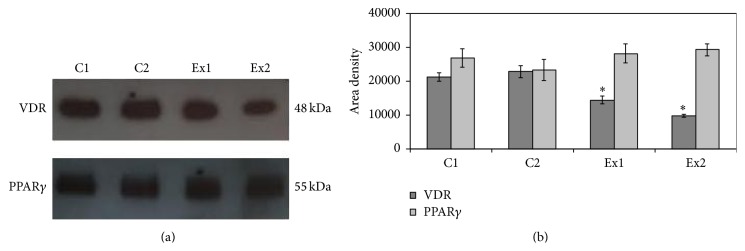
Immunoblotting of vitamin D receptor (VDR) and peroxisome proliferator-activated receptor gamma (PPAR*γ*). (a) Immunoblot of proteins was probed with specific antibodies and visualized by ECL in a patient with anorexia nervosa and normal level of vitamin D3 (C1), a patient with bulimia nervosa and slightly reduced level of vitamin D3 (C2), a patient with anorexia nervosa and very low level of vitamin D3 (Ex1), and a patient with bulimia nervosa and very low level of vitamin D3 (Ex2). Apparent molecular weight was calculated according to the migration of molecular size standards. (b) The area density was calculated with Scion Image programme on densitometry scanning; the data represent the mean ± SD of three experiments performed in duplicate. (Significance, ^*∗*^ *P* < 0.001 versus C1 and C2 sample.)

The results highlighted that the band density of VDR, corresponding to 48 kDa apparent molecular weight, was less colored in Ex samples than in C samples (Figure 2(a)). The analysis of band density showed that VDR of Ex patients was significantly lower than that present in C patients (Figure 2(b)). In particular the VDR Ex1 was reduced 33% and Ex2 55% with respect to that of C patients whose value was very similar.

Since PPAR is regulated by vitamin D, we choose to test PPAR expression by immunoblotting to evaluate an activation of the inflammatory pathway related to the diet. No changes in protein level were found (Figures 2(a) and 2(b)).

**Figure 3:**
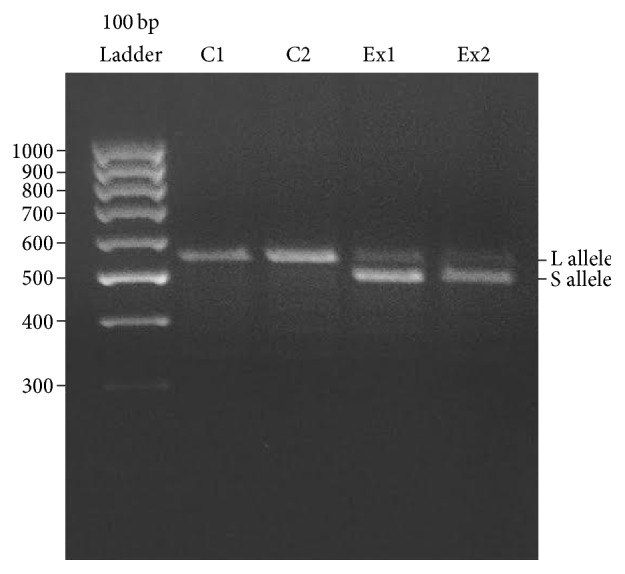
Agarose gel electrophoresis of 5-HTTLPR polymorphism amplified by PCR. The two patients with very low level of vitamin D3, E1, and E2 bear the short allele (S). The two control patients are instead homozygous for the long allele (L). The molecular weight of the corresponding bands is calculated according to the migration of molecular size standards (see Section 2).

The two experimental patients with very low level of vitamin D3 and reduction of VDR had family problems, negativistic and depressive personality disturbances. It was demonstrated that 5-HTTLPR had an impact on the development of eating-disorder symptoms [29] and thus we studied 5-HTTLPR in C and Ex patients chosen for the study. Our results demonstrated the presence of 5-HTTLPR variant short allele (S) in both Ex samples with a very long history of AN (Ex1) and BN (Ex2) that was absent in C patients (Figure 3).

### 3.2. Discussion

We herein reported the case of a woman with long-term AN and the case of a woman with long-term BN complicated by very low level of vitamin D, decrease of VDR, leukopenia, and the S allele of the 5-HT transporter polymorphism. This information emerged from a study of 18 AN (n. 11) and BN (n. 7) patients without blood test profile that suggested endocrine, metabolic, or absorption disorders. Because the goal of the work was to highlight associations of very low amount of vitamin D3 with other parameters in patients with eating disorders, as controls we chose two patients enrolled in the same study, one with AN who had normal levels of vitamin D3 and one with BN who had slightly low value of vitamin D. It was known that vitamin D3 deficiency increases the risk for acquiring several infectious diseases [30] since it had effects on innate and adaptive immunity and has antimicrobial, anti-inflammatory, and immunomodulatory functions [31]. Our data showed that the only two patients who had severe hypovitaminosis presented reduction of WBC. At the moment we do not know if these patients had a defect of immunity response. It will be interesting in the future to investigate the association of immunodeficiencies and severe hypovitaminosis D3 in AN and BN patients. It could be very important because we showed that the blood cells of the two patients with severe hypovitaminosis D3 had a reduced content of the VDR and T cells have been shown to express the VDR and to be both direct and indirect targets of vitamin D3 [32]. We have recently demonstrated that VDR is located in nuclear lipid microdomains that act as platform for active chromatin anchoring so that vitamin D3 can regulate gene expression [33]. So we speculate on the possibility that the very low level of vitamin D3 and the reduction of VDR in blood cells might be responsible for the S allele of the 5-HTT polymorphism that was described to be related to eating disorder, as reported above [21]. On the other hand, the 5-HTTLPR S allele has been associated with differential susceptibility for anxiety and depression in multiple psychiatric disorders [34] and the two AN and BN patients under study presented these symptoms in their personal history. It could be due to the modification of the serotonergic systems that support emotion by 5-HTTLPR S allele that induced reduction of gene and protein expression [17]. In this way the transporter function was less efficient and consequently serotonin remained in the synapse longer and was recycled more slowly, resulting in a net reduction in circulating serotonin [17].

## 4. Conclusions

The most prominent feature of the study was the identification of a woman with long-term AN and a woman with long-term BN with severe hypovitaminosis D3. This study provides a unique insight into the association among the very low level of vitamin D3, the decrease of VDR, leukopenia, and 5-HTTLPR extended to emotional dysfunction. The results induce hypothesising that the severe hypovitaminosis D3 might be responsible for lack of inflammatory response and reduction in mood in patients with long-term eating disorders. The results could be useful to use vitamin D3 as anti-inflammatory molecule for novel therapies in the treatment of eating disorders.

## Figures and Tables

**Table 1:** Parameters considered for the study: ED = eating disorder; TO = time of onset (years); VD3 = vitamin D3 (16–60 ng/mL, normal values); WBC = white blood cells (3.6–9.6, normal values); N = neutrophils (42%–75%, 1.9 × 10^3^–8.0 × 10^3^, normal values); L = lymphocytes (20.5%–51.1%, 0.9 × 10^3^–3.5 × 10^3^, normal values); I = iron (55–150 *μ*g/dL, normal values); F = ferritin (11–307 *μ*g/dL, normal values); T = transferring (180–360 *μ*g/dL, normal values); C = cholesterol (130–220 mg/dL, normal values); G = glycemia (60–110 mg/dL, normal values).

N°	Age	ED	TO	VD3	WBC	N	L	I	F	T	C	G
1	40	BN	<10	**10.4**	8.6	79	15.0	64	50	207	166	72
2	18	AN	<10	27.0	4.38	**37.8**	**52.0**	**51**	74.8	203	139	77
3	19	BN	<10	35.0	7.75	70.5	20.9	126	30.5	313	144	85
4	41	AN	<10	18.6	14.29	67.7	25.4	166	63	294	190	84
5	16	BN	<10	**13.3**	4.19	53.7	36.5	140	59.9	254	146	76
6	55	BN	<10	**14.0**	4.32	56	34.3	91	45.7	231	165	90
7	16	AN	<10	22.0	4.27	60.4	28.8	59	60.7	212	**110**	70
8	40	AN	<10	25.4	5.99	49.1	42.5	119	59.0	272	170	72
9	23	BN	<10	18.1	4.69	66.1	22.5	85	18.8	242	163	72
10	18	AN	<10	19.5	4.66	46.9	43.2	94	183.5	211	147	103
11	15	AN	<10	17.5	4.70	60.0	31.2	98	54	208	213	77
12	18	BN	<10	27.1	6.78	67.9	22.7	**40**	41.6	245	158	71
13	29	**AN**	**15**	**3.60**	**2.91**	**54.4 **	36.6	67	43.1	248	158	82
14	14	AN	<10	20.7	4.37	57.0	32	128	62.8	215	160	76
15	65	AN	<10	30.0	6.62	66.0	22.6	70	81.3	266	157	74
16	44	**BN**	**26**	**4.00**	**3.21**	64.0	**17.0**	82	47.5	212	171	89
17	18	AN	<10	31.6	4.81	47.9	40.7	**48**	65.8	262	226	80
18	18	AN	<10	27.5	4.47	41.1	48.0	60	216	219	133	72
